# Supplementary material for: Kawasaki Disease and Vaccination: Prospective Case-Control and Case-Crossover Studies among Infants in Japan
Source: Vaccines (Basel). 2021 Jul 30;9(8):839. doi: 10.3390/vaccines9080839 (PMC8402330; doi:10.3390/vaccines9080839)
Supplement: Supplementary file 1 [file vaccines-09-00839-s001.zip › vaccines-1256834-supplementary.pdf]

Table S1. Clinical features of patients with Kawasaki disease.

Clinical features	(*n*)	KD
Day of diagnosis (days)[1]	(102)	4 (2–9)
Fever	(102)	102 (100%)
Conjunctival injection	(102)	97 (95.1%)
Erythema of lips and strawberry tongue	(102)	89 (87.3%)
Cervical lymphadenopathy	(102)	74 (72.5%)
Rash	(102)	99 (97.1%)
Erythema and edema of the hands and feet	(102)	95 (93.1%)
Incomplete KD	(102)	8 (7.8%)
Risk score[1,2]	(101)	3 (1–9)
Neutrophil (%)[1]	(101)	57.3 (20.8–81.5)
Platelet count (x10^4/μL)[1]	(101)	40.3 (7.0–99.0)
AST (U/L)[1]	(101)	35 (18–471)
Sodium (mEq/L)[1]	(101)	136 (131–146)
C-reactive protein (mg/dL)[1]	(101)	6.97 (2.05–41.88)
IVIG-refractory	(99)	16 (16.2%)
Coronary artery aneurysms	(102)	14 (12.7%)

n: number of patients, KD: Kawasaki disease, AST: aspartate aminotransferase, IVIG: intravenous immunoglobulin, [1]: median (range), [2]: according to reference [1].

Table S2. Multivariable analysis with patient background and each vaccine as fixed variables.

**(a)**	**OR**	**(95%CI)**	***p***
Age (months)	2.13	(1.32–3.44)	0.002
Sex (male)	0.53	(0.21–1.36)	0.19
Breast feeding	2.32	(0.57–9.50)	0.24
Baby food	2.44	(0.82–7.27)	0.11
History of allergic disease	0.33	(0.09–1.23)	0.10
Symptoms of infection within 2 months before hospitalization	0.96	(0.39–2.34)	0.93
Antibiotic use within 2 months before hospitalization	0.90	(0.28–2.85)	0.85
Sibling(s)	0.60	(0.29–1.26)	0.18
Family history of KD	1.91	(0.48–7.62)	0.36
Housing style (detached house)	1.61	(0.70–3.73)	0.26
PCV13	0.98	(0.32–2.95)	0.97

**(b)**	**OR**	**(95%CI)**	***p***
Age (months)	2.16	(1.33–3.50)	0.002
Sex (male)	0.54	(0.21–1.37)	0.19
Breast feeding	2.41	(0.58–9.99)	0.23
Baby food	2.52	(0.84–7.58)	0.10
History of allergic disease	0.33	(0.09–1.25)	0.10
Symptoms of infection within 2 months before hospitalization	0.96	(0.39–2.35)	0.93
Antibiotic use within 2 months before hospitalization	0.88	(0.28–2.78)	0.83
Sibling(s)	0.58	(0.28–1.21)	0.15
Family history of KD	1.88	(0.47–7.55)	0.38
Housing style (detached house)	1.58	(0.68–3.67)	0.28
HBV vaccine	0.78	(0.28–2.17)	0.64

**(c)**	**OR**	**(95%CI)**	***p***

Age (months)	2.38	(1.43–3.96)	0.001
Sex (male)	0.54	(0.22–1.37)	0.20
Breast feeding	2.64	(0.61–11.53)	0.20
Baby food	2.71	(0.88–8.33)	0.08
History of allergic disease	0.27	(0.07–1.10)	0.07
Symptoms of infection within 2 months before hospitalization	1.00	(0.40–2.47)	1.00
Antibiotic use within 2 months before hospitalization	0.84	(0.26–2.67)	0.77
Sibling(s)	0.53	(0.25–1.10)	0.09
Family history of KD	1.80	(0.43–7.62)	0.42
Housing style (detached house)	1.62	(0.69–3.82)	0.27
DPT-IPV	0.39	(0.14–1.09)	0.07

**(d)**	**OR**	**(95%CI)**	***p***
Age (months)	2.10	(1.30–3.39)	0.002
Sex (male)	0.53	(0.21–1.36)	0.19
Breast feeding	2.28	(0.56–9.21)	0.25
Baby food	2.38	(0.80–7.08)	0.12
History of allergic disease	0.34	(0.09–1.25)	0.10
Symptoms of infection within 2 months before hospitalization	0.95	(0.39–2.33)	0.91
Antibiotic use within 2 months before hospitalization	0.92	(0.29–2.92)	0.88
Sibling(s)	0.62	(0.29–1.30)	0.20
Family history of KD	1.92	(0.48–7.63)	0.35
Housing style (detached house)	1.63	(0.71–3.78)	0.25
Hib vaccine	1.19	(0.37–3.85)	0.77

**(e)**	**OR**	**(95%CI)**	***p***
Age (months)	2.10	(1.31–3.36)	0.002
Sex (male)	0.51	(0.20–1.33)	0.17
Breast feeding	2.38	(0.59–9.66)	0.22
Baby food	2.39	(0.81–7.06)	0.11
History of allergic disease	0.36	(0.10–1.33)	0.12
Symptoms of infection within 2 months before hospitalization	0.96	(0.40–2.33)	0.93
Antibiotic use within 2 months before hospitalization	0.90	(0.28–2.87)	0.86
Sibling(s)	0.64	(0.30–.34)	0.24
Family history of KD	1.91	(0.47–7.72)	0.36
Housing style (detached house)	1.66	(0.72–3.83)	0.24
Rotavirus vaccine	1.32	(0.62–2.77)	0.47

**(f)**	**OR**	**(95%CI)**	***p***
Age (months)	2.27	(1.38–3.74)	0.001
Sex (male)	0.54	(0.21–1.38)	0.20
Breast feeding	2.43	(0.58–10.15)	0.22
Baby food	2.81	(0.91–8.67)	0.07
History of allergic disease	0.31	(0.08–1.18)	0.09
Symptoms of infection within 2 months before hospitalization	0.92	(0.37–2.27)	0.85
Antibiotic use within 2 months before hospitalization	0.89	(0.28–2.81)	0.84
Sibling(s)	0.55	(0.26–1.18)	0.12

Family history of KD	1.87	(0.46–7.60)	0.38
Housing style (detached house)	1.74	(0.74–4.10)	0.21
BCG vaccine	0.58	(0.17–1.96)	0.38

A multivariable analysis was performed with each vaccine (**(a)** PCV13, **(b)** HBV vaccine, **(c)** DPT-IPV, **(d)** Hib vaccine, **(e)** Rotavirus vaccine, or **(f)** BCG vaccine) and 10 fixed variables using conditional logistic regression analysis. OR: Odds ratio, 95% CI: 95% confidence interval, KD: Kawasaki disease, PCV13: 13-valent pneumococcal conjugate vaccine, HBV: Hepatitis B virus, DPT-IPV: Diphtheria, pertussis, tetanus and inactivated poliovirus vaccine, Hib: *Haemophilus influenzae* type b, BCG: *Bacillus Calmette-Guérin*.

Table S3. Case-crossover study limited to the first dose of each vaccine.

	Number of Subjects Who Received Vaccine in the Case Period	Number of Subjects Who Received Vaccine in the Control Period	IR_{MH}	(95% CI)
**(S) 7 days**				
**PCV13**	2	4	**0.50**	(0.09–2.73)
**HBV vaccine**	2	2	**1.00**	(0.14–7.10)
**DPT-IPV**	2	2	**1.00**	(0.14–7.10)
**Hib vaccine**	2	3	**0.67**	(0.11–3.99)
**Rotavirus vaccine**	2	1	**2.00**	(0.18–22.06)
**BCG vaccine**	2	3	**0.67**	(0.11–3.99)
**(L) 28 days**				
**PCV13**	8	14	**0.57**	(0.24–1.36)
**HBV vaccine**	9	11	**0.82**	(0.34–1.97)
**DPT-IPV**	9	9	**1.00**	(0.39–2.52)
**Hib vaccine**	8	14	**0.57**	(0.23–1.36)
**Rotavirus vaccine**	6	9	**0.67**	(0.24–1.87)
**BCG vaccine**	7	17	**0.41**	(0.17–0.99)

IR_{MH}: Mantel–Haenszel incidence ratio, 95% CI: 95% confidence interval, PCV13: 13-valent pneumococcal conjugate vaccine, HBV: Hepatitis B virus, DPT-IPV: Diphtheria, pertussis, tetanus, and inactivated poliovirus vaccine, Hib: *Haemophilus influenzae* type b, BCG: *Bacillus Calmette-Guérin*.

Table S4. Vaccines, adjuvants, and corresponding pattern recognition receptors.

Vaccines	Antigens	Adjuvants		Pattern Recognition Receptors
		Alum	Others	
**Live-attenuated**				
Rotavirus vaccine	Attenuated rotavirus	-		TLR3, MDA5, RIG-I
BCG vaccine	BCG	-	self-adjuvant (CWS, ssRNA, CpG DNA)	TLR2, TLR4, TLR8, TLR9, NODs, CLRs
**Non-live composite**				
PCV13	Polysaccharide, diphtheria toxoid	+		NLRP3, TLR2, TLR4, NOD1 & 2
HBV vaccine	Recombinant HBs antigen	+		NLRP3, TLRs
DPT-IPV	DPT, inactivated polio	+		NLRP3, TLRs
Hib vaccine	OMPC, tetanus toxoid	-		TLR2, TLR4

Data were collected from references [2–5]. BCG: *Bacillus Calmette-Guérin*, PCV13: 13-valent pneumococcal conjugate, HBV: Hepatitis B virus, DPT-IPV: Diphtheria, pertussis tetanus, and inactivated polio vaccine, Hib: *Haemophilus influenzae* type b, OMPC: outer membrane protein complex,

CWS: cell wall skeleton that consists of peptidoglycan, arabinogalactan, and mycolic acids, CLR: C-type lectin receptor, MDA5: melanoma differentiation-associated gene 5, NLR: nucleotide-binding oligomerization domain (NOD)-like receptor, NLRP3: nucleotide-binding oligomerization domain-like receptor family: pyrin domain-containing 3, NOD: nucleotide oligomerization domain, RIG-I: retinoic acid-inducible gene I, TLR: toll-like receptor.

References

1. Kobayashi, T.; Inoue, Y.; Takeuchi, K.; Okada, Y.; Tamura, K.; Tomomasa, T.; Kobayashi, T.; Morikawa, A. Prediction of intravenous immunoglobulin unresponsiveness in patients with Kawasaki disease. *Circulation* **2006**, *113*, 2606–2612, doi:10.1161/CIRCULATIONAHA.105.592865.
2. Philbin, V.J.; Levy, O. Developmental biology of the innate immune response: implications for neonatal and infant vaccine development. *Pediatr Res* **2009**, *65*, 98r-105r, doi:10.1203/PDR.0b013e31819f195d.
3. Pulendran, B.; Ahmed, R. Immunological mechanisms of vaccination. *Nat Immunol* **2011**, *12*, 509–517, doi:10.1038/ni.2039.
4. Holloway, G.; Coulson, B.S. Innate cellular responses to rotavirus infection. *J Gen Virol* **2013**, *94*, 1151–1160, doi:10.1099/vir.0.051276-0.
5. Saso, A.; Kampmann, B. Vaccine responses in newborns. *Seminars in immunopathology* **2017**, *39*, 627–642, doi:10.1007/s00281-017-0654-9.
